# A sympathetic view of blood pressure control at high altitude: new insights from microneurographic studies

**DOI:** 10.1113/EP089194

**Published:** 2020-12-20

**Authors:** Lydia L. Simpson, Craig D. Steinback, Mike Stembridge, Jonathan P. Moore

**Affiliations:** ^1^ Institute for Sport Science Division of Physiology Innsbruck University Innsbruck Austria; ^2^ Neurovascular Health Laboratory Faculty of Kinesiology Sport, and Recreation University of Alberta Edmonton Canada; ^3^ Cardiff School of Sport and Health Sciences Cardiff Metropolitan University Cardiff UK; ^4^ Extremes Research Group School of Sport Health and Exercise Sciences Bangor University Bangor UK

**Keywords:** blood pressure control, high altitude, microneurography, muscle sympathetic nerve activity

## Abstract

**New Findings:**

- **What is the topic of the review?**
  Sympathoexcitation and sympathetic control of blood pressure at high altitude.
- **What advances does it highlight?**
  Sustained sympathoexcitation is fundamental to integrative control of blood pressure in humans exposed to chronic hypoxia. The largest gaps in current knowledge are in understanding the complex mechanisms by which central sympathetic outflow is regulated at high altitude.

**Abstract:**

High altitude (HA) hypoxia is a potent activator of the sympathetic nervous system, eliciting increases in sympathetic vasomotor activity. Microneurographic evidence of HA sympathoexcitation dates back to the late 20th century, yet only recently have the characteristics and underpinning mechanisms been explored in detail. This review summarises recent findings and highlights the importance of HA sympathoexcitation for the regulation of blood pressure in lowlanders and indigenous highlanders. In addition, this review identifies gaps in our knowledge and corresponding avenues for future study.

## Graphical abstract

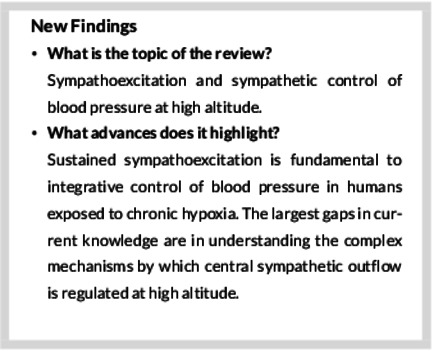

## 1 INTRODUCTION

High altitude (HA) exposure is a significant stressor of cardiovascular function and autonomic regulation. Acutely, activation of the peripheral chemoreceptors increases heart rate and cardiac output as a means to preserve systemic oxygen delivery. Acute hypoxic exposure also increases sympathetic vasomotor outflow to the skeletal muscle vasculature (muscle sympathetic nerve activity; MSNA) (Saito et al., 1988) to restrain local vasodilator mechanisms (Weisbrod et al., 2001). With extended exposure to HA, several time‐dependent acclimatisation processes occur, including ventilatory and haematological alterations, which progressively restore arterial oxygen content ($C_{aO_{2}}$) and normalise cardiac output to sea‐level values (Sutton et al., 1988). Paradoxically, HA acclimatisation is not accompanied by normalisation of MSNA.

In the first published microneurographic study at HA, Duplain et al., (1999) reported an augmented MSNA burst frequency following 24–36 h at 4559 m. This was greater than that observed during 20 min exposure to an equivalent hypoxic stimulus ($F_{IO_{2}}$ 0.12) in the same participants, which suggested MSNA is augmented in response to increasing durations of exposure. Subsequently, Hansen and Sander, (2003) reported a 200% increase in MSNA burst frequency following 4 weeks’ residence at 5260 m, which occurred alongside normalisation of $C_{aO_{2}}$. Furthermore, elevated MSNA persisted for 3 days following descent to sea level. These seminal studies were followed by a 15‐year hiatus in published HA microneurographic data. Recently, however, a number of studies have presented new data, including the first MSNA recordings in highland native populations, and address the mechanisms underpinning HA sympathoexcitation. This recent work provides the primary focus for this Hot Topics Review.

## 2 SYMPATHETIC NEURAL ACTIVITY AT HA

**FIGURE 1:**
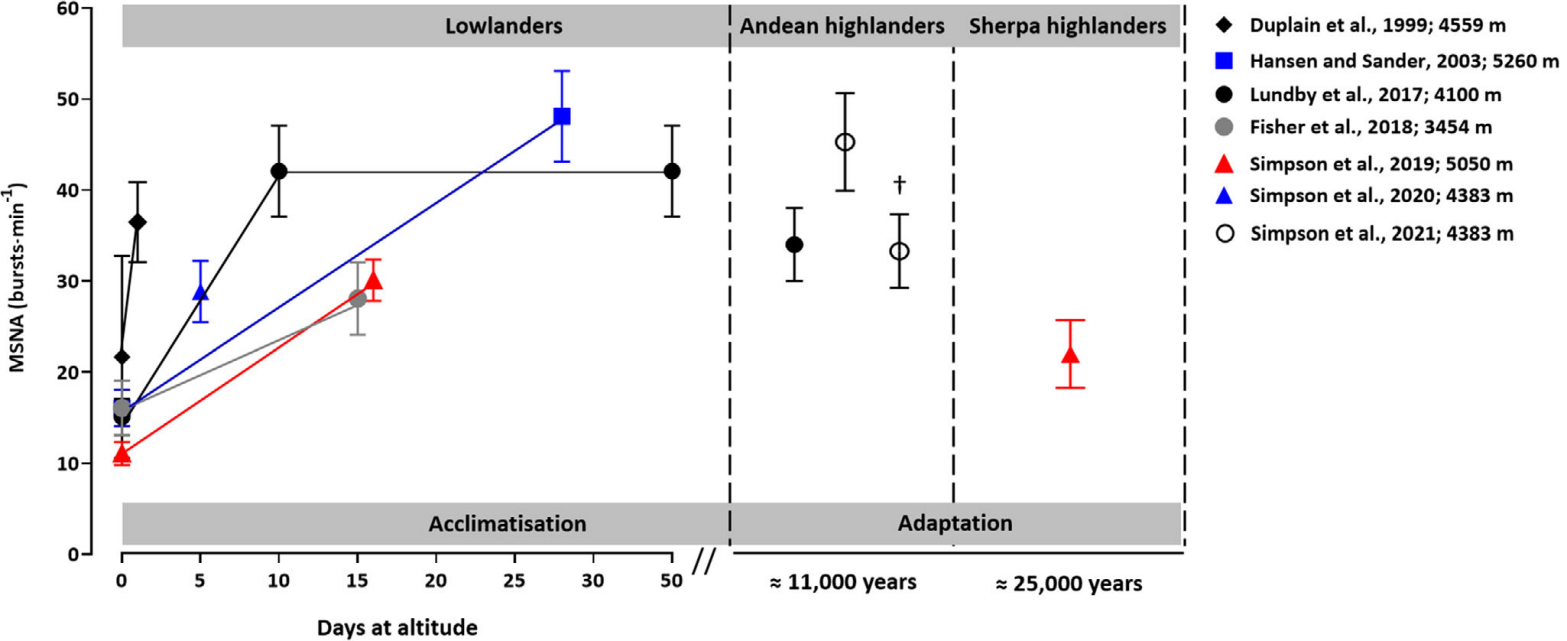
Summary of the changes in muscle sympathetic nerve activity (MSNA) burst frequency during acclimatisation in lowlanders and adaptation in highland natives. Values presented as means ± SEM. †Andeans with chronic mountain sickness

Sustained sympathoexcitation in lowlanders during HA acclimatisation has been confirmed by ourselves and others (Fisher et al., 2018; Lundby et al., 2017; Simpson et al., 2019). Furthermore, it is evident that heightened sympathetic vasomotor outflow is mediated by increases in both burst frequency and burst size (Busch et al., 2020; Simpson et al., 2019), and the extent of HA sympathoexcitation is influenced by the severity of hypoxic stress (Fisher et al., 2018; Table 1). In the only serial measurements recorded at HA, Lundby et al., (2017) reported a 180% increase in MSNA burst frequency in lowlanders following 10 days at 4100 m (15 vs. 42 bursts min^–1^), with no further change following 50 days, providing important temporal information regarding the MSNA response at HA. The large inter‐individual variability in basal MSNA and sympathetic responsiveness to hypoxia, in addition to the different ascent profiles and between‐laboratory variability in analysis methods, make direct comparisons between studies difficult. Nevertheless, in newly exposed lowlanders, MSNA appears to progressively increase during the initial days at HA, plateau within 10 days of exposure, and is maintained for the duration of exposure, at least up to 50 days (Figure 1). This is noteworthy, as ventilatory, haematological and cardiac responses to HA are dynamic across the acclimatisation process, whereas sympathoexcitation is persistent.

**TABLE 1:** Summary of microneurographic studies in lowlander populations at HA

Microneurographic study	Participants	Ascent method	Exposure duration	Altitude studied at	Magnitude of change MSNA burst frequency	MAP, mean ± SEM (mmHg)	Magnitude of change in MAP
Duplain et al., (1999)	Seven male lowlanders (39–41 years)	Passive/active	24–36 h	4559 m	69% increase	Not reported	—
Hansen & Sander, (2003)	Four male, four female lowlanders (24 ± 2 years)	Not reported	28 days	5260 m	200% increase	LA: 77 ± 2, HA: 87 ± 3	14% increase
Fisher et al., (2018)	Nine male, one female lowlanders (26 ± 4 years)	Passive	15–17 days	3454 m	75% increase	LA: 82 ± 4, HA: 91 ± 3	12% increase
Lundby et al., (2017)	Six male, two female lowlanders (22–31 years)	Passive	10 days	4100 m	180% increase	LA: 72 ± 2, HA: 78 ± 2	8% increase
	As above	As above	50 days	4100 m	180% increase	LA: 72 ± 2, HA: 75 ± 2	4% increase
Simpson et al., (2019)	12 male, two female lowlanders (27 ± 6 years)	Active	10–20 days	5050 m	173 % increase	LA; 84 ± 2, HA; 85 ± 3	NS

Due to large inter‐individual variability in basal MSNA at sea level, the relative change in MSNA for lowlanders at HA versus sea level are displayed. HA, high altitude; LA, low altitude; MAP, mean arterial pressure; MSNA, muscle sympathetic nerve activity; NS, non‐significant.

Despite acclimatisation, lowlanders exhibit a limited ability to adapt to HA. In contrast, highland indigenous populations have adapted over many generations, with divergent adaptation strategies apparent between populations (Beall, 2006). For example, natives of the South American Andes primarily rely on increased haemoglobin levels, and thus high $C_{aO_{2}}$, to maintain oxygen delivery to tissues. In contrast, Himalayan Sherpa appear to rely on enhanced peripheral blood flow and downstream metabolic adaptations to meet oxygen demand (Erzurum et al., 2007; Horscroft et al., 2017). Sympathetic neural activity, which is an important regulator of systemic and peripheral blood flow, may contribute to these phenotypical differences. However, until recently, very little was known regarding sympathetic neural activity in highlanders, due to the lack of microneurographic data.

Lundby and colleagues (2017) reported comparable MSNA burst frequencies in Bolivian Aymara Andean highlanders and lowlanders following 10 days at 4100 m, which was two‐fold higher than that of lowlanders at sea‐level (Figure 1). In contrast, Sherpa exhibit MSNA burst frequencies that are 73% lower than those observed in lowlanders following 10–20 days at HA. (Simpson et al., 2019; Figure 1). Furthermore, MSNA burst frequency is 70% lower in Sherpa, when compared with values obtained in Andean highlanders of a similar age and body mass index (Lundby et al., 2017). Strikingly, this is despite Andeans been studied at a lower altitude (4100 vs. 5050 m) and exhibiting greater oxygen saturations (87 vs. 82%). Whilst several factors, independent of genotypic differences, can influence MSNA (i.e., physical activity, diet and environmental factors) (Notay et al., 2017), these findings indicate that divergent pathways of adaptation extend to the sympathetic neural response to HA. In other words, Sherpa have adapted to favour a lower sympathetic vasomotor outflow than Andeans. Whilst the benefits of lower sympathetic vasomotor outflow are unclear, this may facilitate augmented peripheral blood flow (Erzurum et al., 2007), and protect against the potentially negative vascular consequences associated with chronically elevated sympathetic vasomotor activity (Malpas, 2010). Nevertheless, MSNA for Sherpa at 5050 m was ∼30% greater than that observed for Sherpa at 1440 m (Simpson et al., 2019), indicating that HA hypoxia remains a potent sympathetic stressor, despite evolutionary adaptation.

The majority of highland natives demonstrate successful adaptation to the HA environment; however, a small percentage of individuals lack the ability to adapt, and develop the maladaptation syndrome chronic mountain sickness (CMS). CMS, which is most prevalent (>15%) in Andean highlanders (Leon‐Velarde et al., 2005), is characterised by an excessive haematological response to ambient hypoxia (haemoglobin concentration ≥21 g/dl for men, ≥19 g/dl for women) and exaggerated arterial hypoxaemia for the resident altitude (Leon‐Velarde et al., 2005). Excessive erythrocyte volume, and the resulting elevations in haematocrit and blood viscosity, increase resistance to blood flow, exacerbate ventilation–perfusion mismatch and impair pulmonary gas exchange (Winslow et al., 1985), which further exacerbates arterial hypoxaemia. Despite lower arterial oxygen tensions and saturations, Simpson et al (2021) reported a 25% lower MSNA burst frequency in Andeans with mild CMS, compared to healthy Andean highlanders (Figure 1). Whether this is also true for more severe cases of CMS remains unclear and a direction for future study.

Whilst it must be acknowledged that these studies involve small samples, sympathetic activation appears to be a feature of HA exposure in both lowlanders and highlanders. However, duration of altitude residence, elevation, ascent profile, ethnicity and presence or absence of CMS likely influence the degree of sympathoexcitation. There may be a fundamental regulatory role for heightened MSNA at HA, which is to maintain systemic vasomotor tone and arterial pressure.

## 3 ARTERIAL BLOOD PRESSURE REGULATION AT HA

### 3.1 Baroreflex control of sympathetic vasomotor activity

The arterial baroreflex is the preeminent short‐term controller of arterial blood pressure, primarily via beat‐to‐beat changes in sympathetic vasomotor outflow and i.e. MSNA. Observations that heightened MSNA (between 75% and 200%) is accompanied by a small elevation in resting arterial pressure (4–14%; Table 1) led to a suggestion that baroreflex control of MSNA is impaired by chronic exposure to ambient hypoxia (Fisher et al., 2018; Hansen and Sander, 2003; Lundby et al., 2017). This hypothesis was recently tested, utilising pharmacologically induced changes in blood pressure (modified Oxford test) (Simpson et al., 2019). However, whilst exposure to acute normobaric hypoxia ($F_{IO_{2}}$ 0.11) reduced vascular sympathetic baroreflex responsiveness, there was no evidence that vascular sympathetic baroreflex responsiveness (i.e., gain) was different at 5050 m (10–20 days’ exposure) compared with low altitude (LA; 344 m). Furthermore, vascular sympathetic baroreflex gain for Sherpa was comparable with lowlanders, and resting arterial blood pressure was remarkably similar for both groups at HA and for lowlanders at LA. Despite similarities in baroreflex gain and arterial pressure, the vascular sympathetic baroreflex operated at a different MSNA operating point (i.e., MSNA burst incidence). For lowlanders, the MSNA operating point was higher at 5050 m compared with LA, indicating an upward resetting of the vascular sympathetic baroreflex. For Sherpa, the vascular sympathetic baroreflex operating point was lower than for lowlanders at 5050 m, yet higher than for lowlanders at LA.

Using the same approach, vascular sympathetic baroreflex function has also been explored in Peruvian Andeans of Quechan ancestry, with and without CMS (Simpson et al., 2021). Unexpectedly, vascular sympathetic baroreflex responsiveness was similar in both groups. In addition, reflex gains were similar to those observed previously for lowlanders and Sherpa (Simpson et al., 2019). Furthermore, despite considerable variability in basal MSNA across populations, arterial pressure was similar for lowlanders, Sherpa and Andeans. Collectively, these findings indicate that the ability of the baroreflex to regulate MSNA is not impaired under the physiological stress of HA hypoxia, even with the presence of CMS. In fact, the vascular sympathetic baroreflex is equally effective at buffering beat‐by‐beat fluctuations in arterial pressure among lowlanders and highlanders, at least during moderate blood pressure challenges (±15 mmHg above and below resting arterial pressure). Nevertheless, the vascular sympathetic baroreflex operates with a higher MSNA operating point to maintain arterial pressure at HA. Whether the higher operating point limits the vasoconstrictor response to initial orthostatic hypotension, due to a reduced sympathetic reserve, remains to be determined.

In addition to sympathetic vasomotor outflow, arterial blood pressure is also determined by local vascular control mechanisms, cardiac output and blood volume (Guyenet, 2006), which all change, to varying degrees, in response to HA exposure. It is, therefore, important to examine the shift in baroreflex MSNA operating point and elevated basal MSNA in the context of these other factors.

### 3.2 Vascular control mechanisms

Augmented MSNA counteracts vasodilatory mechanisms during systemic hypoxia (Weisbrod et al., 2001) and may offset blunted neurovascular transduction (i.e., vasoconstrictor response to a burst of MSNA). Indeed, Berthelsen et al., (2020) reported a blunted pressor response following a burst of MSNA in lowlanders at HA, compared to LA. Moreover, Sherpa exhibit a greater pressor response to a burst of MSNA at HA, whereas Andeans exhibit a smaller pressor response compared to both lowlanders and Sherpa at HA. Furthermore, in the same groups of participants, Busch et al (2020) reported a greater pressor response to MSNA in Sherpa during maximal apnoea, although this was not replicated during isometric handgrip.

Down‐regulation of cardiac β‐adrenergic receptor sensitivity is observed during chronic HA exposure in lowlanders (Richalet et al., 1988), and similar adrenergic desensitisation or decreased responsiveness may occur within the vasculature, mediating the reduced neurovascular transduction. Simpson et al., (2019) reported that an identical dose of an α_1_‐adrenergic receptor agonist, phenylephrine, elicited a lower pressor response following 10–20 days at 5050 m compared to 344 m, indicating a reduced α_1_‐adrenergic receptor sensitivity. Moreover, in Sherpa at 5050 m, a similar pressor response was elicited by a smaller dose of phenylephrine, compared to lowlanders at 5050 m. Overall, augmented MSNA in lowlanders at HA is possibly a compensatory response to blunted neurovascular transduction, and population differences in basal MSNA, without substantial variation in resting arterial pressure, may reflect differences in vascular sensitivity to MSNA. Nevertheless, pharmacological investigations would be required to determine this possibility. Furthermore, it remains to be determined whether, in fact, neurovascular transduction is blunted as a response to elevated MSNA.

### 3.3 Circulating blood volume

It has previously been suggested that sympathetic vasomotor outflow is augmented in lowlanders at HA to compensate for a reduction in circulating blood volume (Hansen and Sander, 2003). Blood volume depletion, secondary to reductions in plasma volume, begins within 24 h at HA and serves as an adaptive response to increase haemoglobin concentration early during exposure (Ryan et al., 2014). Despite progressive expansion of erythrocyte volume after 7 days at altitude (Ryan et al., 2014), blood volume remains reduced during the first 4 weeks of exposure (Siebenmann et al., 2017). Acute reductions in blood volume exert an excitatory influence on sympathetic vasomotor outflow, via engagement of the arterial baroreflex, with the observation of an inverse relationship between these factors in healthy males at sea level (Best et al., 2014). Indeed, briefly restoring blood volume, via 1000 ml saline infusion, elicits a 20% reduction in MSNA burst frequency in lowlanders following 4 weeks at 5260 m (Hansen and Sander, 2003). Nevertheless, despite restoration of blood volume, MSNA remained significantly elevated above sea level values (∼150%), suggesting other important contributory mechanisms. Stembridge et al., (2019) demonstrated a greater total blood volume in Sherpa, compared to acclimatising lowlanders, at 5050 m, with Sherpa also exhibiting lower MSNA (Simpson et al., 2019). Moreover, Andeans with mild CMS exhibit a 20% greater total blood volume, secondary to an increased erythrocyte volume, compared to healthy Andeans (Claydon et al., 2004; Simpson et al., 2021). This greater blood volume corresponded to 25% lower MSNA, leading to a conclusion that relative sympathoinhibition in mild CMS balances haemodynamic effects of excessive erythrocytosis (i.e., elevated blood volume and blood viscosity) and maintains arterial pressure within a normal range (Simpson et al., 2021). Nevertheless, direct manipulation of blood volume is required to investigate its role in MSNA regulation in highlanders.

In summary, arterial blood pressure at HA is determined by integration of several physiological factors. An upward shift in baroreflex MSNA operating point and ensuing sympathoexcitation appear to be part of an integrated response to HA exposure, which work to maintain both oxygen and arterial pressure homeostasis. Indeed, the absence of such elevations in MSNA at HA, particularly in recently exposed lowlanders, could lead to hypotension, decreasing perfusion of vital organs and exacerbating an already compromised oxygen delivery.

## 4 MECHANISMS INVOLVED IN NEURAL ADJUSTMENTS TO HA

The mechanisms underlying the neural adjustments to HA, which likely involve alterations in peripheral feedback and central regulatory mechanisms, have not been interrogated fully. The remaining section considers the mechanistic contribution of autonomic reflexes originating from peripheral chemoreceptors and pulmonary arterial baroreceptors to vascular sympathetic baroreflex resetting and sympathoexcitation at HA.

### 4.1 Peripheral chemoreceptors

Carotid body sensitisation and enhanced translation of chemoreceptor afferent input to the central nervous system are responsible for a time‐dependent increase in ventilation in lowlanders exposed to chronic HA hypoxia (i.e., ventilatory acclimatisation). The ventilatory and sympathetic chemoreflex share a common afferent pathway (Guyenet, 2000); thus, it is reasonable to consider a role for peripheral chemoreceptors in sympathoexcitation during sustained HA hypoxia (Dempsey et al., 2014). Nevertheless, attempts to acutely eliminate peripheral chemoreflex drive at HA indicate the effect on basal MSNA is modest or even minimal. For example, Hansen & Sander (2003) reported that 25 min of hyperoxia reduced basal MSNA by 7 bursts min^–1^ (∼20%), following 4 weeks’ acclimatization at 5260 m. Notably, sympathetic vasoconstrictor drive remained substantially elevated compared to sea level values (∼175%). Following a shorter sojourn (10–20 days) at 5050 m, minimal change in MSNA burst frequency and total activity were observed when breathing 100% O_2_ for 5 min (Simpson et al., 2019). Furthermore, there was no change in vascular sympathetic baroreflex function (i.e., MSNA operating point, operating pressure and gain). Similarly to lowlanders, hyperoxia does not significantly influence vascular sympathetic baroreflex function in Andean highlanders nor Nepalese Sherpa, and has minimal effects upon basal MSNA at HA (Simpson et al., 2019; Simpson et al., 2021). In this study, hyperoxia duration was relatively brief, in order to eliminate peripheral chemoreceptor drive whilst minimising secondary effects of changes in CO_2_ and minute ventilation. Fisher et al. (2018) also observed that blunting carotid chemoreceptor drive, via low‐dose intravenous dopamine infusion, did not change MSNA in lowlanders after 15–17 days at 3454 m; however, systemic effects of dopamine complicate the interpretation of these findings. Nevertheless, it is evident that whilst the peripheral chemoreflex appears to be central in initiating sympathoexcitation during acute hypoxic exposures, the peripheral chemoreflex is not solely responsible for sustained sympathoexcitation during HA acclimatisation and adaptation. Importantly, there appears to be a dissociation between ventilatory and sympathetic responses during chronic hypoxia, a notion that is supported by recent studies of peripheral chemoreflex activation during acute hypoxia (Keir et al., 2019; Prasad et al., 2020). Nevertheless, the relative contribution of the peripheral chemoreflex mechanism to basal MSNA appears to vary between lowlanders, Andeans and Sherpa, which may be influenced by ancestral differences in peripheral chemoreceptor sensitivity to hypoxia (Beall et al., 1997; Severinghaus et al 1966).

### 4.2 Pulmonary arterial baroreceptors

**FIGURE 2:**
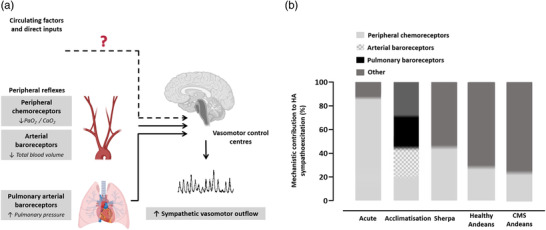
Mechanisms for high altitude (HA) sympathoexcitation. Alterations in afferent input to several peripheral reflexes contribute to elevated basal MSNA at HA, as shown in (a). The approximate contributions of each peripheral reflex mechanism, based on the studies described in section 2 and 3, are shown in (b). Additional mechanisms contributing to HA sympathoexcitation may include alterations in circulating factors and direct inputs to the sympathetic vasomotor control centres. CMS, chronic mountain sickness

In response to ambient hypoxia, the pulmonary vascular smooth muscle contracts, elevating pulmonary arterial vascular resistance and pressure (Groves et al., 1987). Notably, the seminal work by Duplain et al., (1999) reported a strong, positive relationship between pulmonary arterial pressure (PAP) and MSNA burst frequency. Furthermore, in normoxic anesthetised animals, isolated increases in PAP elicit increases in sympathetic outflow in a positive feedback manner, via stimulation of baroreceptors located in the extra pulmonary arteries (Moore et al., 2011). The potential sympathoexcitatory role of pulmonary arterial baroreceptors in humans at HA has since been investigated (Simpson et al., 2020). During inhalation of vasodilator nitric oxide, which selectively lowered PAP by 25%, a similar reduction in MSNA burst frequency was observed in lowlanders at 4383 m (exposure range 4–9 days). This reduction in MSNA corresponded to a downward and leftward resetting of the vascular sympathetic baroreflex. These findings support a hypothesis that afferent feedback from pulmonary arterial baroreceptors contributes to resetting of baroreflex control of MSNA and sympathoexcitation at HA. Additionally, incomplete reversal of PAP elevations following return to LA (Groves et al., 1987; Maufrais et al., 2016) may contribute to maintained elevations in MSNA following descent (Hansen and Sander 2003; Mitchell et al., 2018). It is less evident how pulmonary arterial baroreceptors might contribute to sympathetic control of blood pressure for indigenous high‐altitude populations. However, it is noteworthy that Sherpa, for whom basal MSNA is lower compared with lowlanders and Andeans at HA, display minimal hypoxic pulmonary hypertension (Groves et al., 1993). Therefore, differences in PAP may contribute to the differences in vascular sympathetic baroreflex MSNA operating point and basal MSNA between populations. Similarly to other mechanisms investigated, the magnitude of the reduction in MSNA during reductions in PAP was relatively modest (Figure 2). It is possible, however, that a greater reduction in PAP (i.e., to sea level values) would have elicited a greater reduction in MSNA.

Overall, HA exposure is accompanied by alterations in afferent input to several peripheral reflexes, including those originating from the peripheral chemoreceptors, arterial baroreceptors (in response to a reduction in blood volume) and pulmonary arterial baroreceptors (in response to elevated PAP). These autonomic reflexes all contribute to sustained sympathoexcitation at HA (Figure 2). Whilst the mechanistic contribution of these reflexes appear to vary with exposure duration, and may be influenced by ancestry, they account for ∼70% of the increase in basal MSNA (Figure 2) in acclimatising lowlanders. The mechanisms accounting for the remaining ∼30% of HA sympathoexcitation require investigation, with candidate mechanisms including alterations in central nitric oxide bioavailability, systemic inflammation and oxidative stress (Lewis et al., 2014), central erythropoietin production (Oshima et al., 2018), and alterations in intracranial pressure (Schmidt et al., 2018) (Figure 2). Moreover, whilst the contribution of the peripheral chemoreflex to HA sympathoexcitation in highlanders has been examined, the relative contribution of additional mechanisms requires investigation.

## 5 CONCLUSION

Sympathoexcitation is a feature of HA exposure, not only in lowlanders, but also in highland natives who have generational exposure to ambient hypoxia. A shift to a higher baroreflex MSNA operating point permits elevated MSNA at HA, whilst maintaining the ability to buffer acute changes in arterial pressure. Elevated MSNA is a functional adaptation, compensating for alterations in vascular control mechanisms and blood volume, which protect against hypotension, and maintain oxygen demand at HA, especially in lowlanders. These neural adjustments likely involve a complex integration of multiple mechanisms, whose contribution may change as a function of duration of HA exposure. Indeed, the peripheral chemoreflex appears to be the primary mechanism responsible for the initiation of elevated sympathetic vasomotor outflow during acute hypoxic exposures; however, additional mechanisms, including alterations in blood volume and afferent input from pulmonary arterial baroreceptors, contribute to augmented sympathetic vasomotor outflow during sustained exposures. Despite these recent advances, future studies are necessary to determine the precise physiological mechanisms by which chronic HA hypoxia promotes sympathetic vascular baroreflex resetting and elevated sympathetic vasomotor outflow. Moreover, greater female inclusion is required for examination of potential sex differences in sympathetic neural regulation at HA in both lowlander and highland native populations.

## COMPETING INTERESTS

None.

## AUTHOR CONTRIBUTIONS

Conception and design: L.L.S., J.P.M., M.S. and C.D.S. Drafting and revision of the manuscript L.L.S., J.P.M., M.S. and C.D.S. All authors approved the final version of the manuscript and agree to be accountable for all aspects of the work in ensuring that questions related to the accuracy or integrity of any part of the work are appropriately investigated and resolved. All persons designated as authors qualify for authorship, and all those who qualify for authorship are listed.
